# Supplementary material for: Exploring the Antibacterial Potential of Konjac Glucomannan in Periodontitis: Animal and In Vitro Studies
Source: Medicina (Kaunas). 2023 Oct 6;59(10):1778. doi: 10.3390/medicina59101778 (PMC10608271; doi:10.3390/medicina59101778)
Supplement: Supplementary file 1 [file medicina-59-01778-s001.zip › medicina-2615864-supplementary.pdf]

Supplementary Materials

To measure the minimum inhibitory concentration (MIC), each well of 96 well plates was mixed with 100 µL of a BHI broth medium, 5 µL of *P. gingivalis*, 5 µL of KGM at different doses (10, 8, 6, 4, 3, 2, 1, 0.5, 0 5 µg/mL), and 5 µL of the triphenyltetrazolium chloride reagent. The positive control consisted of 1 µg/mL of azithromycin (AZT), while the negative control consisted of 5 µL of dimethylsulfoxide (DMSO). The well plate was incubated for 24 h and filled with CO_2 gas at a temperature of 37 °C in the incubator. The color change was observed visually by looking at the pink color that is formed. The clearer the color indicates the absence of bacterial growth, which is the lowest concentration of the MIC value. Furthermore, the OD value was observed before and after incubation. The MIC was found in the positive control group (1 µg/mL AZT), as shown in Supp. Figure S1.

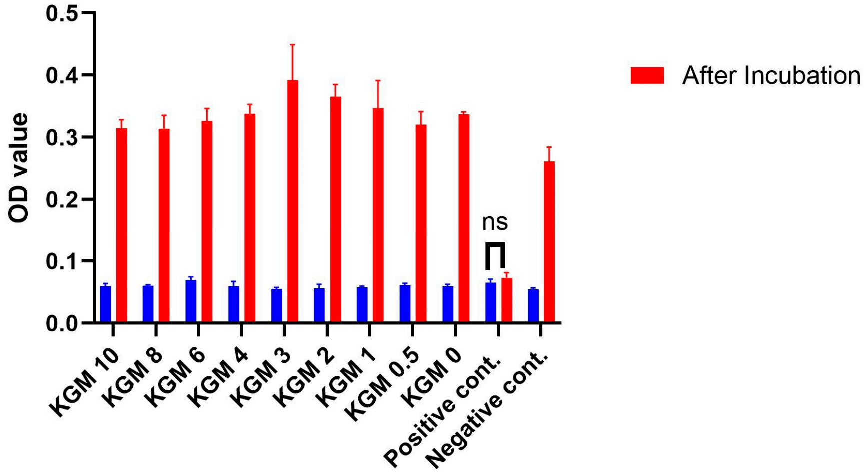

Figure S1. The paired t-test shows no significant differences of OD values in the positive control group (AZT).

To analyze Minimum Bactericidal Concentration (MBC), 100 µL of *P. gingivalis* and 100 µL of BHI media were added into the petri dish and then homogenized by rotating the petri dish to form a figure of eight. Then, a paper disc was placed using tweezers on each petri dish, and 10 µL of KGM at different doses (10, 8, 6, 4, 3, 2, 1, 0.5, 0 5 µg/mL) were added and incubated at 37 °C for 24 h. The diameter of the inhibition zone was measured in millimeters (mm) using a digital caliper. The MBC was found in the positive control group (1 µg/mL AZT) with a diameter of 12 mm (as shown in Supp Figure S2).

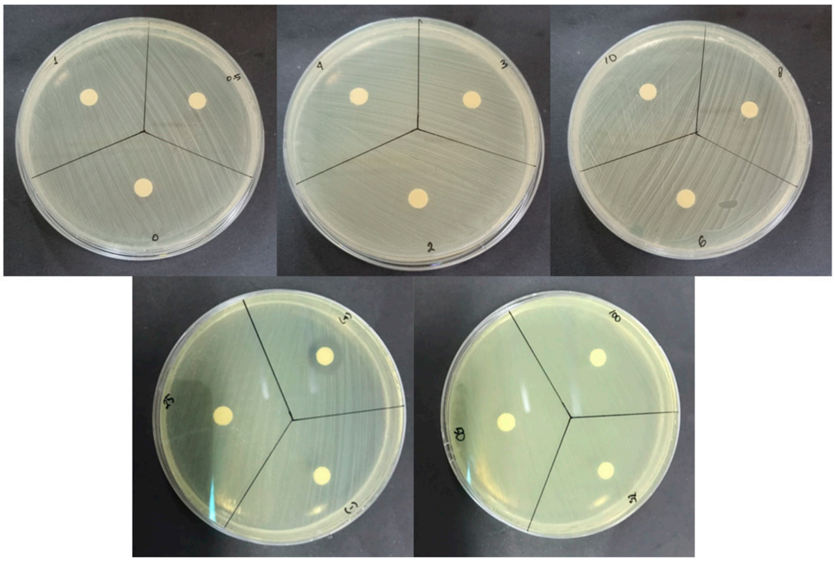

Figure S2. The inhibition zone was formed in the positive control group (AZT).

Table S1. Alveolar bone loss evaluation from histology (in vivo).

**Group (n = 3)**	**Alveolar bone loss Mean ± SD (mm)**	***p* value**
Control	0.08167 ± 0.02401	<0.0001 **
KGM	0.09167± 0.01722	
Periodontitis	0.3150 ± 0.09311	
KGM + Periodontitis	0.1950 ± 0.06473	

Uji ANOVA *one way*; ** $p \leq 0{,}01$; SD: Standard deviation.
